# Mpox in Africa: What we know and what is still lacking

**DOI:** 10.1371/journal.pntd.0013148

**Published:** 2025-06-12

**Authors:** Jia Bainga Kangbai, Umaru Sesay, Ulrich Vickos, Fatmata Kagbanda, Mosoka Papa Fallah, Augustus Osborne

**Affiliations:** 1 Department of Public Health, Njala University, Bo City, Sierra Leone; 2 Sierra Leone Field Epidemiology Training Program, National Public Health Agency, Freetown City, Sierra Leone; 3 African Field Epidemiology Network, Freetown City, Sierra Leone; 4 Laboratory of Arbovirus, Haemorrhagic Fevers, Institute Pasteur de Bangui, Emerging Virus and Zoonosis, Bangui, Central African Republic; 5 Africa Centres for Disease Control and Prevention, Addis Ababa, Ethiopia; 6 Department of Biological Sciences, Njala University, Bo City, Sierra Leone; NIAID Integrated Research Facility, UNITED STATES OF AMERICA

## Abstract

Emerging as a major global health threat, Mpox previously known as Monkeypox has drawn attention due to a worrying surge in cases. This zoonotic disease, native to Central and West Africa, is marked by fever, rash, and lymphadenopathy and is primarily spread through direct contact with infected animals or people and indirectly through contaminated objects. Recent studies have indicated possible sexual transmission, underscoring how human behavior and environmental changes are increasing its prevalence, even though human-to-human transmission is less efficient than that of smallpox. Mpox is endemic in several African countries, and currently, the infection has spread in non-endemic countries, including Rwanda, Uganda, and Kenya. Democratic Republic of Congo is the epicenter of the current Mpox outbreak. From January 1, 2022, to August 6, 2024, sixteen African countries reported Mpox outbreak. Several factors, including population immunity deficiencies and changes to the environment and ecology, have led to the widespread of Mpox in Africa. Challenges such as the fragile healthcare system, limited vaccine availability and access, weak surveillance, and low public awareness poses difficulty in containing the infection in affected countries. Given the potential of Mpox to disrupt several sectors including health systems, which may ultimately reverse progress in achieving the sustainable development goals by 2030. It is imperative for countries, both within and outside Africa, to extend financial aid and human resources to combat the infection effectively.

## Introduction

Historically known as Monkeypox, Mpox is an endemic zoonotic disease mostly found in parts of Central and West Africa and is characterized by fever, rash, and lymphadenopathy, among other symptoms. Mpox was first identified in 1958 [1,2]; the first human case was reported in 1970 in the Democratic Republic of Congo (DRC) [3]. Since that time (1970s), there have been sporadic outbreaks with an increasing trend in incidence and geographical spread over the past months. The Mpox pathogen is primarily transmitted through direct contact with infected animals or humans and indirectly by contact with fomites [4–6]. In recent times, there have been reports of sexual transmission of Mpox [7–9]. Human transmission of Mpox is inefficient compared to smallpox, but changes in human behavior and environmental factors make such a transmission pattern more common. The role of animal reservoirs, especially rodents and small primates, is crucial in preventing the transmission of the Mpox virus in nature. However, there remains limited evidence on the role of animal reservoirs and sexual transmission in spreading Mpox. Understanding the specific animal species that act as reservoirs is crucial for predicting and preventing spillover events. Additionally, clarifying the mechanisms and prevalence of sexual transmission is essential for developing targeted public health interventions. Addressing these gaps will enhance our ability to control and eventually eliminate Mpox in affected countries and by extension regions.

## Current Mpox situation in Africa compared with other regions

**Fig 1:**
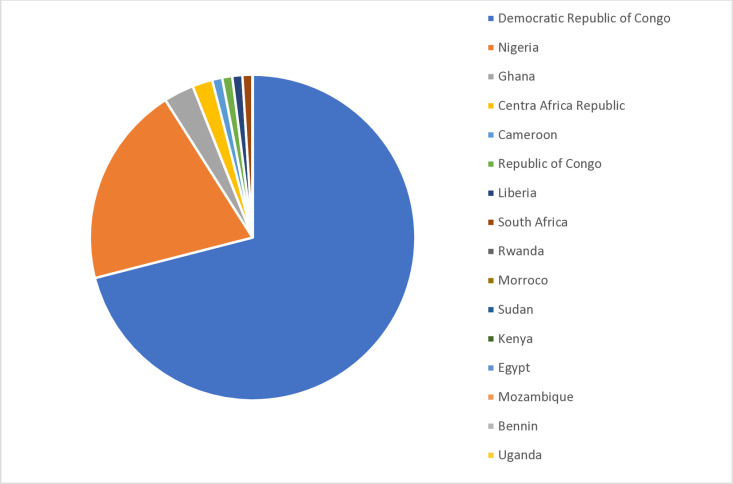
Distribution of Mpox cases in Africa, 2022–August 2024.

Mpox is endemic in several African countries. From January 1, 2022, to August 6, 2024, sixteen African countries reported laboratory-confirmed Mpox virus cases (Fig 1) [10], with a total of 2030 confirmed cases and 13 deaths [7–9]. There have never been any historically reported Mpox case for many of these countries, including Burundi, Kenya, Rwanda, and Uganda. To date, African countries have recorded more confirmed and suspected Mpox cases (a total of 17,500) in 2024 than in 2023 (a total of 15,000) [11,12]. The DRC is the epicenter of the current Mpox outbreak in Africa [13], and the majority of the Mpox cases in Africa in 2024 so far have been children ≤15 years. In the DRC, this sub-population accounts for two-thirds of Mpox incidence [14]. The spread of the virus in these countries is largely blamed on the ongoing conflict in the region, which has resulted in the mass displacement of civilians across their different borders. Other critical challenges to curtailing the Mpox outbreak in Africa include limited healthcare infrastructures compounded with limited medical supplies and professionals, community beliefs in seeking healthcare from traditional healers, and limited funding [15]. Worth noting that Ghana has no history of Mpox outbreaks; however, the country was identified in 2003 as the source of a shipment of wild mammals that led to Mpox outbreak in US that year.

In September 2023, a new Mpox variant (clade 1b) emerged in the eastern DRC [14] which shares borders with Rwanda and Uganda. Rwanda, Uganda, and most recently, Kenya have reported cases of the new Mpox variant [11]. Four countries: Burundi, Kenya, Rwanda, and Uganda with no previous outbreaks of Mpox have reported cases since mid-July 2024. Epidemiological data from these countries indicates an increasing trend of Mpox cases in the past few months; this increase may be attributed to environmental and ecological changes, improved surveillance, and a waning level of population immunity following the cessation of smallpox vaccination programs.

Compared to other regions globally (Table 1), like Europe, specifically the United Kingdom, ranks in the eighth position worldwide regarding the burden of Mpox [16]. Most cases occur among men who identify as gay or bisexual or men who have sex with men, with transmission mainly occurring through close skin contact [16]. This pattern highlights the importance of targeted public health measures and awareness efforts to curb the spread within these communities. Similar patterns were also observed in North America, like Canada, where most of the cases occur among men who have sex with men, and the primary route of transmission is reported through sexual intercourse [17].

**Table 1:** Differences in the epidemiology of the African Mpox outbreak with other regions.

Dimensions	Africa	Other regions
Epidemiology	Predominantly zoonotic, with sporadic outbreaks linked to wildlife exposure.	Human-to-human transmission is more prevalent, particularly among specific communities.
Transmission	Primarily through contact with infected animals and consumption of bushmeat.	Close human contact, including sexual contact, is the primary mode of transmission.
Affected population	Rural communities with proximity to wildlife.	Men who have sex with men (MSM) and urban populations.
Public health interventions	Focus on wildlife management and educating communities about risks.	Targeted awareness campaigns and vaccination strategies for high-risk groups.
Challenges	Limited healthcare infrastructure and surveillance systems.	Stigma and misinformation affecting intervention efforts.
Vaccination	Limited access to vaccines; reliance on preventive measures.	Availability of vaccines and implementation of vaccination campaigns.
Surveillance	Often reactive, with challenges in timely outbreak detection.	More robust systems in place for monitoring and response.

## Current public health response

### Vaccination

The global Mpox response varies by country, with the majority focusing on vaccination, contact tracing, quarantine, and public education. Most Mpox vaccination campaigns and activities revolve around the use of the smallpox vaccine (ACAM2000) and the newer MVA-BN vaccine. Despite the success of using smallpox vaccination to prevent the spread of Mpox elsewhere. In Africa, the large geographic landscape coupled with vaccine hesitancy, culture, the presence of armed conflict, low vaccine supply, and other logistical challenges involving the supply chain, pose a major challenge to Mpox vaccination effort.

### Contact tracing and quarantine

Contact tracing and quarantine of cases are the commonly used strategy implemented by African countries to control and manage the spread of Mpox outbreaks. However, like the case of the vaccination effort, contact tracing and quarantine are also being hampered by many challenges, including a weak healthcare infrastructure, insufficient funds, and the vast and remote geographical areas of the affected regions.

### Public awareness

Various African countries and organizations have launched campaigns, and awareness-raising activities to educate their communities about Mpox, mostly targeting the disease transmission mode, prevention strategies, early recognition of its symptoms, and the significance of seeking early treatment. However, like the prevention of most diseases in Africa, misinformation and cultural beliefs continue to pose significant barriers to these public awareness efforts.

### International collaboration and support

Recently, the Africa Centers for Disease Control and Prevention (Africa CDC) and the World Health Organization have collaboratively implemented strategic initiatives to enhance public health response capabilities in the African continent. These initiatives include establishing a unified framework titled “One budget, one plan, and one monitoring and evaluation framework,” alongside creating the “One Continental Incident Management Support” [18]. Through these coordinated efforts, Africa CDC is actively disseminating routine updates on outbreak situations, which facilitates informed decision-making and enhances awareness regarding epidemiological trends and patterns of Mpox across member states.

Additionally, these initiatives have led to the successful procurement of 20,000 mpox cartridges to bolster diagnostic testing capabilities throughout member states. In response to the acute needs in Burundi and the DRC, which are among the most affected countries, Africa CDC, in partnership with UNICEF, has allocated $58 million USD in humanitarian aid aimed at supporting children and marginalized groups [19]. These comprehensive measures underscore the commitment to strengthening health systems and mitigating the impacts of ongoing outbreaks in the region.

## Challenges and opportunities

### Challenges

- **Healthcare infrastructure:** Many African countries lack the robust healthcare infrastructure needed for effective Mpox surveillance, diagnosis, and treatment. To compound this problem, most of the areas severely affected by Mpox in Africa are remote regions; rural and remote areas are mostly underserved in Africa.
- **Public awareness:** Like most other diseases with outbreak potential, there is a low level of public awareness about Mpox which contributes to its delayed diagnosis and treatment. Low public awareness increases the risk of severe treatment outcomes and viral transmission.
- **Access to vaccines:** One of the greatest challenge to the ongoing control and management of the Mpox outbreak in Africa is the limited access to smallpox vaccines, both in terms of supply and distribution. Also, because Clade 1 Mpox strains are sexually transmissible, identifying adults at risk for Mpox vaccination. One last mile for accessing the Mpox vaccine effort in African countries is how to design vaccine programs to target at-risk children. In the current Mpox outbreak in Africa, the children at risk are mostly ≤5 years old, which will require the design of Mpox vaccine program for children <2 years. This is coupled with the likelihood that the parents of those at-risk children will accept vaccination for their kids given the non-existence of data on the use of such vaccine in this subpopulation.
- **Need for epidemiological study:** There remains a gap in knowledge about the growing trend of Mpox in the current outbreak that requires further investigation. There is a growing incidence of childhood Mpox cases being reported in the current outbreak, thus making the need to investigate the source of childhood Mpox transmission.
- **Socioeconomic, political, and cultural factors:** Multiple studies have highlighted several factors deterring the containment of the ongoing Mpox outbreak. In the DRC, research has identified specific socio-cultural and environmental determinants. The predominant occupation of residents as farmers, alongside traditional practices involving rodent hunting among youth and children, overcrowded living conditions, and communal use of household utensils, significantly increase the risk of Mpox transmission [20–22]. Additionally, Olawade and colleagues reported that deforestation, agricultural expansion, urbanization, and climate change are critical factors exacerbating human- wildlife interactions, potentially leading to zoonotic transmission of Mpox in Africa [23]. The study emphasized that climatic changes alter rainfall patterns and ecological conditions, influencing the distribution of animal reservoirs and the Mpox virus, thereby facilitating outbreaks [23]. In Nigeria, a study reported that low political support, characterized by chronic underfunding, misadministration, and high healthcare costs, impedes successful Mpox containment efforts [24]. McNab and colleagues further noted the neglect by political leaders in providing necessary funding, logistics, treatment, and vaccines as major barriers to containment efforts in the African region [25]. The authors advocated for increased political attention and leadership at national, regional, and global levels, urging member states and the WHO to implement International Health Regulations (IHR), increase funding, supply vaccines, and enhance community engagement as strategies to curb the spread [25].
- **Limited funding:** The absence of dedicated funding for the Mpox response presents a major challenge to the implementation of essential prevention and control measures. Currently, several African countries lack a dedicated financial support for outbreak management. In instances where funding is available, it is often insufficient to effectively sustain response activities. Consequently, this pose difficulties in proactively implementing outbreak preparedness and response interventions necessary to timely halt or mitigate the spread of the infection.

### Opportunities

- **Strengthening surveillance:** The topmost priority in the control and management of the current Mpox outbreak in Africa is the enhancement of disease surveillance, especially in rural areas. This could lead to earlier detection of cases, leading to a robust and effective outbreak response.
- **Capacity building:** African countries and their international partners should invest in their healthcare infrastructure, increase training of public health personnel, and provide the crucial resources that will improve their ability to respond to Mpox outbreaks in real-time to stop it spread.
- **International collaboration:** The fight to stop the expansion of the current Mpox outbreak in Africa should not be an African struggle. The current Mpox outbreak and its expansion call for African countries to establish new partnerships and collaborations, as well as extend existing ones. This will enable their governments to facilitate the sharing of resources, expertise, and vaccines that will invariably lead to improving the overall response to mpox in Africa.

## Global implications

The limited vaccine availability and access, low public awareness, movements of people, and poor healthcare infrastructure within African countries make it difficult to curtail the infection. This poses a high risk of transmission globally. The widespread Mpox infection would not only result in high mortality and morbidity but would also disrupt several other activities including travel restrictions and slowing economic growth. Considering the weak immunity of children, becoming infected with Mpox as seen in the affected African countries, would increase their vulnerability to many other infections. Consequently, this will result in a high fatality rate, thereby reducing the continent’s future generation with a high potential of contributing to economic growth within Africa and beyond. The ongoing conflict in many countries globally poses a high risk of Mpox transmission, as evidenced in Burundi, Kenya, Rwanda, and Uganda. The emergence of Mpox in those countries will be extremely fatal, especially since their healthcare system is disrupted by the ongoing war. With the rapid spread of Mpox across the African continent, non-endemic countries must enhance their preparedness measures to prevent the importation of the Mpox virus. Key strategies include training healthcare workers, particularly those in border communities, on Mpox case detection and providing logistical support alongside establishing isolation units at designated health facilities, particularly in border communities. Similar to the COVID-19 pandemic, the spread of Mpox in non-endemic countries could increase the vulnerability of populations, especially children, to vaccine-preventable diseases and limit access to essential medical services, thereby elevating morbidity and mortality rates. Furthermore, the outbreak may reduce the productivity of healthcare workers, who would be overburdened with the provision of essential healthcare services.

## Policy recommendations

Breaking the chain of Mpox transmission in Africa requires a multipronged approach. African countries should heighten public awareness campaign activities, solicit support for Mpox vaccines, train healthcare workers on Mpox case definitions, and enhance surveillance. Governments should provide funds to their respective public health institutes/agencies to heighten preparedness and response efforts. To ensure the swift distribution of Mpox vaccines, African countries should prioritize procurement and mandate vaccination for high-risk groups, including healthcare workers. Integrating Mpox vaccination into routine immunization programs is essential, with a focus on optimizing supply and cold chain systems, particularly in rural areas. The deployment of community health workers (CHWs) is crucial for effective vaccine distribution in remote regions and for enhancing social mobilization to boost vaccine uptake. Moreover, funding from government and international partners should support public health researchers in conducting groundbreaking studies to investigate the transmission patterns of Mpox related to sexual intercourse and identify the virus’s reservoir. Qualitative research in countries without known Mpox transmission is needed to understand the factors behind successful prevention, which can inform strategies to prevent future outbreaks in both endemic and non-endemic regions. Finally, a community-focused care framework is recommended, emphasizing the involvement of CHWs and stakeholders like religious and traditional leaders, youth, and women’s groups. Leveraging local resources and knowledge through this framework enhances vaccine distribution and social mobilization, which are crucial for controlling the outbreak and preventing further transmission.

## Conclusion

Children make up the majority of those infected with Mpox, which is endemic in several African countries and has a high fatality rate. Several factors, including population immunity deficiencies and changes to the environment and ecology, have led to the widespread of Mpox in the African continent. Challenges such as the fragile healthcare system, limited vaccine availability and access, weak surveillance, and low public awareness make it difficult for the containment of the infection in affected countries. Because Mpox has the potential to disrupt the functioning of every sector globally, non-affected countries within and beyond Africa must provide support (financial and human resources) to fight against the infection.

## Abbreviations

**:**
DRC: Democratic Republic of Congo
Mpox: monkey pox
MSM: men who have sex with men
